# From case counts to probability sampling: Simulation insights into pandemic surveillance

**DOI:** 10.1016/j.puhip.2026.100766

**Published:** 2026-03-11

**Authors:** Inken Siems, Ralf Münnich

**Affiliations:** Trier University - Department of Economic and Social Statistics, Universitätsring 15, 54296, Trier, Germany

**Keywords:** Public health surveillance, COVID-19 surveillance, Prevalence estimation, Probability sampling

## Abstract

**Objectives:**

To identify data strategies that ensure valid epidemic surveillance across different prevalence levels.

**Study design:**

A simulation study using realistic microdata from the German MikroSim project, reflecting the demographic structure of two districts. Epidemic dynamics were modeled over one year with an SIR framework, varying prevalence (>0% to 12%), test accuracy (80% to 98%), and sample size (5000 to 30,000).

**Methods:**

Passive case-based surveillance relying on reported infections was compared with probability-based population sampling for estimating weekly prevalence levels and changes.

**Results:**

Case-based surveillance was reliable only at very low prevalence. Once prevalence exceeded 3–5%, estimates became unstable and systematically biased, reflecting testing patterns rather than true infection dynamics. Probability sampling, in contrast, produced unbiased, precise estimates and enabled timely integration of individual-level social and health data.

**Conclusion:**

Surveillance systems should be adaptive. While passive reporting may suffice at low prevalence by practicability and costs, probability-based sampling becomes essential once moderate prevalence thresholds are crossed (3-5%). Such thresholds vary by disease and are shaped by symptom profile and transmission dynamics. Embedding predefined prevalence-based switch points that trigger representative sampling would ensure valid estimates, strengthen preparedness, and support timely, evidence-based public health decision-making.

## 1 Introduction

Accurate estimates of infection prevalence are essential for effective disease control, especially in pandemics. During the COVID-19 pandemic, however, many countries relied on legacy surveillance systems, based on physician-reported diagnoses, voluntary testing, and laboratory confirmations. These systems suffer from behavioral selection, access disparities, and incomplete coverage [1], which, in statistical terms, constitutes non-probability sampling. As the virus spread, the limitations of such passive surveillance grew more apparent, particularly in capturing asymptomatic and mildly symptomatic cases.

These biases were not merely theoretical. Seroprevalence studies revealed that reported case counts frequently underestimated true infections by factors of five to ten [2,3]. As prevalence increased, distortions due to selective testing, reporting delays, and structural inequalities became more pronounced, undermining the reliability of epidemic curves derived from routine data. Despite these limitations, such figures shaped public understanding and guided policy. While health authorities asserted their statistics were reliable [4], independent assessments identified substantial inaccuracies [5].

Representative surveillance offers a methodological alternative [6]: probability sampling, which produces unbiased estimates and enables the quantification of uncertainty. During COVID-19, probability-based studies in the UK and Spain demonstrated its value: in the UK, randomized testing detected resurgent spread missed by official counts [7]; in Spain, national serosurveys revealed widespread underreporting [3]. Nonetheless, such efforts remained limited, and most countries continued to rely on convenience data—without clarity when that strategy would fail.

**Fig. 1:**
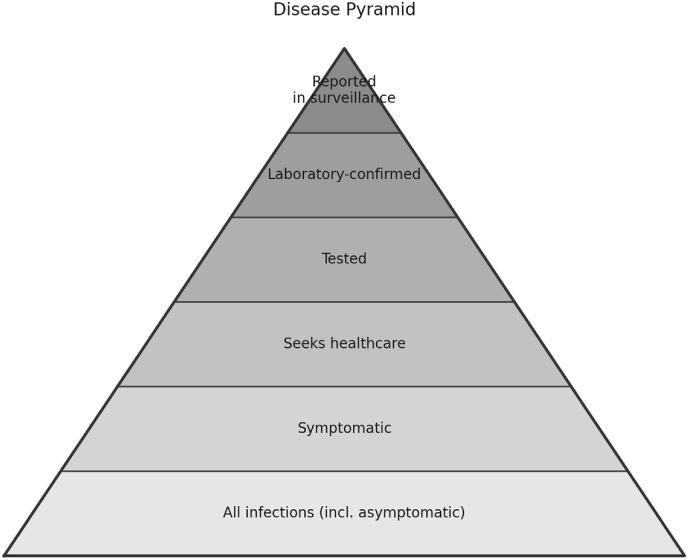
Disease Pyramid shows the attrition from community infections to reported cases. Asymptomatic and mild infections are least likely to be detected.

The present study addresses this critical gap: *At what point must surveillance shift from rapid but biased data streams to probability-based methods?* Using an in-depth simulation of a COVID-19–like epidemic in a synthetic population, we compare the accuracy and bias of two concurrent surveillance strategies—passive (non-probability) and representative (probability-based)—across epidemic phases. Each strategy captures a different extreme of the disease pyramid [8] (Fig. 1): passive surveillance concentrates on clinically detected cases, whereas representative surveillance reflects the full spectrum of infections, including mild and asymptomatic infections.

Our results show that passive surveillance can detect isolated cases at very low prevalence. When prevalence exceeds a modest threshold (approximately 3–5%), non-probability estimates become systematically biased and unstable. Above this point, only probability sampling yields valid estimates of prevalence and change. These findings highlight the need for adaptive surveillance systems that integrate probabilistic sampling based on survey methods as epidemic conditions evolve. There is no substitute for high-quality data when prevalence hangs in the balance.

## 2 Methods

### 2.1 Simulation framework

The simulation is based on realistic microdata from the German MicroSim Project [9], representing private households in two administrative districts of the Trier region. This subset forms a highly accurate statistical digital twin of the area and serves as the reference population for all simulation experiments. Each individual was assigned attributes relevant to SARS-CoV-2 dynamics, including socioeconomic status (GISD) [10], spatial neighborhood classification, weekly infection status, symptom presence, and test outcomes. Epidemic trajectories were modeled using an adapted susceptible-infectious-recovered (SIR) framework allowing for reinfection over 52 weeks, encompassing initial emergence, exponential growth, peak, and decline phases. Further details on the data-generating processes are provided in the Supplementary Material.

### 2.2 Surveillance strategies

• *Non-Probability Sampling (*S_nps*):* This approach emulated traditional passive case reporting at the top of the disease pyramid (Fig. 1), where test likelihood was contingent on symptom presence and GISD. We modeled three testing patterns: constant, tri-phasic waves, and monotonically increasing trends. Prevalence was estimated via a naive sum estimator (H_naive) and an adjusted Generalized Regression (GREG) estimator [11] with propensity weights [12].
• *Probability Sampling (*S_p*):* This method used a four-wave rotational panel design with municipality-stratified proportional allocation. It enables valid population inference and yields population-level estimates representing the bottom of the disease pyramid (Fig. 1). To assess performance under changing epidemic and operational conditions, we evaluated variants differing in second-stage sampling, sample size, test qualities, and testing behaviors (see Table 1). We applied the Horvitz-Thompson (HT) estimator [13] and the GREG estimator [11]. Weekly change rates (CH) were calculated using a ratio estimator [14].

**Table 1:** Simulation scenario parameters.

Dimension	Scenarios
S_p& S_nps	
Quantities Estimated	*POS*, *PREV*
Estimation Levels	Overall, Municipality
Test Qualities	a = 98.1%, b = 98.7%; a = 80%, b = 90%; True values: a = 80%, b = 90%; Applied: a = 98.1%, b = 98.7%

S_p	
Sampling Designs	simple random sampling without replacement (SRS); Spatial SRS; Stratified SRS (GISD, SpN, Building Size Class, Household Size, Age); Cluster (1-stage, 2-stage); Balanced Sampling; πps (inhabitants, GISD, SpN)
Sample Sizes (regional Sampling Fractions)	5000 (<0.1 – 3%); 10,000 (1 – 4%); 20,000 (2 – 5%); 30,000 (3 – 6%)
Drawing Intervals	Weekly, Monthly, Bi-monthly
Estimators	HT, GREG, Change Rates

S_nps	
Testing Patterns	Constant, Tri-wave, Monotonic Increase
Estimators	Naive Sum, Propensity-weighted GREG, Change Rates

Details from sampling and estimation strategies can be drawn from standard textbooks in survey sampling [11,15].

### 2.3 Prevalence estimation

We computed weekly test positivity (*POS*) as the number of RT-PCR–positive results, which is biased for true prevalence due to imperfect tests. We therefore estimated prevalence (*PREV*) using the Rogan–Gladen estimator [16]:

$$\bar{Y}=\frac{{\bar{Y}}^{*}+b-1}{a+b-1}$$

where ${\bar{Y}}^{*}$ is the observed test outcome, and a, b are test sensitivity and specificity. This estimator is asymptotically unbiased under constant test characteristics.

### 2.4 Simulation workflow

An overview of all simulation scenarios is provided in Table 1. The procedure is outlined in Table 2. We assume ideal conditions: unlimited resources, no budget constraints, and no operational limitations.

**Table 2:** Algorithm: Epidemic surveillance simulation.

Step	Description
1	Require: simulation runs R=1000; population of interest; simulation period T=52 weeks.
2	For r=1 to R
3	For week t=1 to 52
4	Draw S_nps and compute naive estimates.
5	If *t* is a sampling week
6	Draw S_p according to specified designs.
7	Calculate HT, GREG and CH estimates.
8	Calculate S_nps GREG.
9	End If
10	End For
11	End For
12	Output: Performance Measures (e.g., bias, MSE, coverage) for *POS* and *PREV*.

## 3 Results

Our study reveals substantial challenges inherent to epidemiological statistics. Sampling in epidemiology is more complex due to additional layers of uncertainty beyond classical sampling frameworks. Estimation uncertainty is often high and dependent on evolving conditions, such as prevalence dynamics, test quality, and heterogeneous testing behavior. These difficulties are further compounded by practical constraints that were not addressed in this simulation study.

### 3.1 Non-probability sample

Non-probability surveillance collapses in accuracy at moderate to high prevalence level. As prevalence increases, point estimates become increasingly biased in magnitude. When true prevalence is low, both over- and under-estimation are common. The adjusted estimator (H_corrected) improves performance but remains sensitive to model misspecification. Deviations between the adjustment model and the true selection mechanism inflate both bias and variance, particularly when auxiliary margins are estimated rather than known.

Use of outdated adjustment data or incorrect test characteristics introduces further error. Differences across the three testing regimes are minor, but higher testing rates tend to amplify bias and the model efficiency of H_corrected depends on the realized number of tests. Change estimates approximate *POS* trends only crudely.

Overall, non-probability data lacks inferential validity. They reflect testing behavior rather than true infection dynamics and cannot support reliable epidemiological conclusions once prevalence surpasses a moderate threshold.

### 3.2 Probability sample

Probability-based sampling maintained statistical validity above moderate prevalence. Design differences were minor and declined with increasing simulation runs and sample size. Stratified simple random sampling yielded the most efficient estimates. Alternative designs, such as cluster sampling, were less efficient, exhibiting higher variation and right-skewed sampling distributions. In particular, optimized designs, such as πps sampling, are highly sensitive to their assumptions, complicating meaningful implementation.

*PREV* estimates showed lower bias and better mean squared error than *POS*, despite being less efficient. The bias of *POS* estimates increased with lower test accuracy and false positives dominated at low true prevalence. Low prevalence and poor test quality produced zero-inflated and skewed sampling distributions. Estimate stability improved with increasing true prevalence and larger samples but remained unreliable below moderate prevalence levels - indicating a need for sufficiently high true prevalence to achieve reliable inference. The gap between *POS* and *PREV* estimates widened with increasing sample size.

**Fig. 2:**
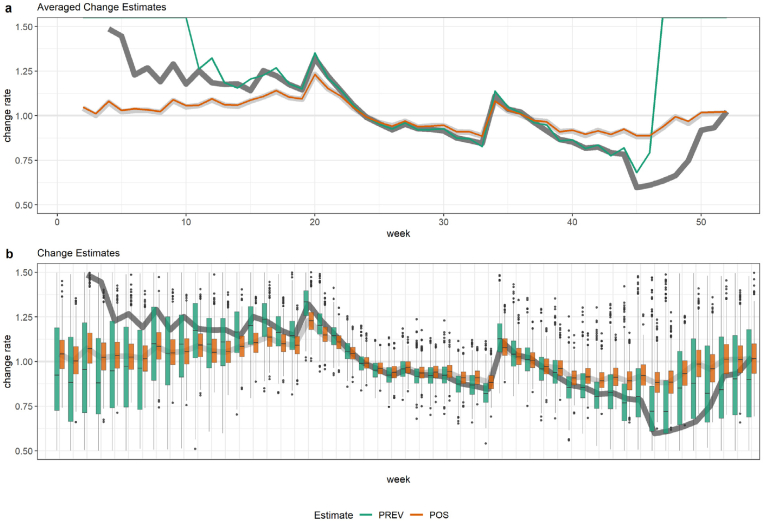
Change estimates are unbiased in expectation but display increased variance near inflection points and at low prevalence. The dark grey line represents the true prevalence change rate, the light grey line the true positivity change rate, while the green and orange lines depict the estimated prevalence (*PREV)* and positivity (*POS)* rates, respectively.

Likewise, change estimates were particularly sensitive to both factors and showed maximum instability at epidemic inflection points (Fig. 2). Across all scenarios, *PREV* change rates exceeded *POS* changes in magnitude. Under conditions of lower test accuracy and high true prevalence, they continued to approximate the true change. However, when incorrect test parameters were applied, both *PREV* and *POS* estimates reflected changes in positivity rather than true prevalence. Variance patterns followed similar trends.

*PREV* coverage of 95% confidence intervals, defined as the proportion of intervals containing the true value, was generally close to nominal levels (mean 90–93%), while *POS* estimates showed near-zero coverage. With increasing sample size, the *PREV* coverage rates of the prevalence estimates decrease in variation, while *POS* increased. *PREV* change coverage had a high median (97%) but lower mean (90%) coverage, indicating instabilities dependent on sample size and prevalence rate. *POS* change estimates reached only 73%, declining further with larger samples. When incorrect test parameters were applied, coverage collapsed for all estimators.

**Fig. 3:**
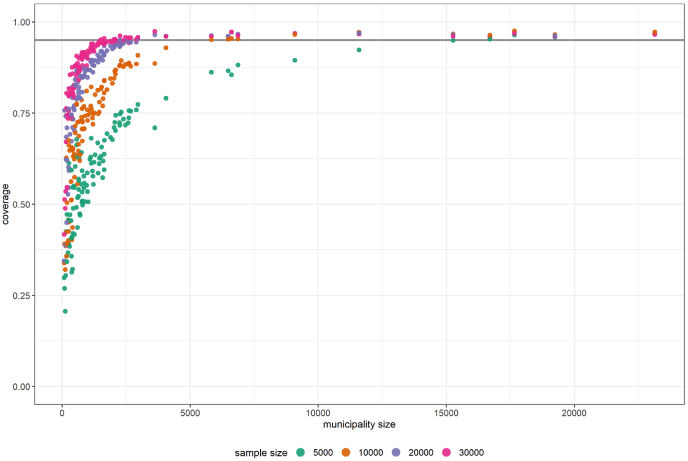
Regional 95% confidence interval coverage rate depends strongly on sample and municipality size. Coverage below the nominal 95% level (grey line) indicates undercoverage, implying that reliable direct regional estimation requires large samples.

Regional patterns mirrored overall trends but with amplified variability. Confidence interval coverage was highly sensitive to design and sample size. Balanced sampling improved regional, but not overall, estimates. Larger municipalities showed near-perfect coverage, while smaller ones required substantially larger samples (Fig. 3). Even with a total sample size of n=10,000, regional CI coverage deteriorated rapidly because proportional allocation produces very small within-municipality subsamples, particularly in smaller municipalities. Under these conditions, local coefficients of variation frequently exceeded 1.5, making direct regional estimates unreliable. This limitation could be addressed by Small Area Estimation (SAE) techniques using e.g., area-level or unit-level models that link direct municipality-week estimates to auxiliary covariates (e.g., demographics) and borrow strength across municipalities and weeks [17]. Such workflows are already used in official statistics and public-health reporting for local indicators, indicating that integration as a post-processing layer on top of probability-based surveillance is feasible [18,19].

## 4 Discussion

Although this study focused on COVID-19, the underlying principle generalizes: Reliable epidemic surveillance requires data that accurately captures the scale and structure of infection. Our results underscore a central finding: When prevalence is low, passive surveillance may suffice. However, as disease prevalence increases, the utility of passive surveillance declines, and once it rises above a critical prevalence threshold (3–5%), passive surveillance systems produce biased and unstable estimates, even with correction. Then, probability sampling becomes essential for valid inference. While the critical threshold may vary by disease features and epidemic progression, the fundamental rationale holds. Operationally, this implies switching surveillance systems.

Passive surveillance can perform well with respect to timeliness, stability, completeness, sensitivity, and representativeness under appropriate conditions [20,21]. However, its evaluation is necessarily retrospective, implying that data quality is unknown at the time of reporting, use and decision-making. Moreover, validation typically relies on health-insurance reimbursement data, and therefore conditions observation on health-care–seeking behavior. This induces a selection mechanism that is external to the disease process itself. Consequently, passive reporting can provide accurate data for diseases with distinctive clinical manifestations that reliably trigger medical consultation, such as measles or Ebola. As these systems are operationally efficient and cost-effective, they are also attractive tools for diseases at low prevalence, particularly for detecting rare or sporadic events.

For diseases characterized by substantial asymptomatic infection, mild symptomatology, or deliberate avoidance of health care, bias becomes systematic and not correctable once prevalence exceeds moderate levels. In such regimes, the bias is structural: it is not identifiable from passive data alone and cannot be eliminated by calibration. While adjustment methods may reduce bias, they rely on unverifiable assumptions, such as conditional ignorability, and typically introduce additional variance. Our findings show that correction methods cannot salvage inference when core data mechanisms are flawed. Empirical evidence from South Korea during the COVID-19 pandemic illustrates this limitation, demonstrating reduced sensitivity of passive surveillance [20,21].

Moreover, attempts to improve inference within the existing frameworks—such as redesigning traditional sentinel surveillance as a probability-based cluster sample with mandatory reporting—do not resolve this limitation. Infected individuals who do not seek health care remain systematically unobserved. As a result, estimates are inherently biased with respect to the target population and typically exhibit higher sampling variance than alternative stratified with direct population sampling. Inference from such monitoring systems is therefore restricted to the upper levels of the disease pyramid (Fig. 1).

In contrast, probability sampling provides unbiased estimates, enables valid comparisons over time and space, and allows adjustment for nonresponse. Accurate prevalence estimation requires rigorous statistical design, reliable test performance, and adaptation to behavioral and structural dynamics. Its implementation requires significant planning: sample size, true prevalence, test qualities, nonresponse patterns, estimator choice, and sampling design, all influence reliability. Among our scenarios, stratified SRS yielded the most robust and efficient results, aligning with the UK's Infection Survey approach [22]. Infections vary in their surveillance demands. For example, COVID-19 posed extraordinary inferential challenges due to rapid spread, policy shifts, behavioral heterogeneity, asymptomatic cases, and structural clustering across networks, geography, and socioeconomic gradients [23].

*PREV* estimates are more accurate but less efficient than *POS*. Misapplication of test characteristics distorts estimates even if epidemic shape can remain recognizable. Local precision is difficult to achieve; even with n=30,000, many municipalities had inadequate coverage. Increasing further the sample size, small area estimation and optimized allocation may help.

Nevertheless, probability-based surveillance has limitations in applicability. At low prevalence or when detecting rare, isolated cases, representative sampling requires prohibitively large sample sizes, yields high variance, and imposes substantial resource demands, rendering it impractical. Change estimators are volatile, especially at epidemic inflection points.

The COVID-19 pandemic exposed critical data gaps, as many socioeconomic indicators—such as employment conditions, household income, and access to social support—remained unmeasured in real time. High-frequency household surveys with a modular design could have addressed these gaps by enabling the timely integration of relevant topic modules. Given the logistical and financial constraints of launching new data infrastructures during a crisis, such platforms provide a pragmatic, scalable approach to capturing the multidimensional impacts of public health emergencies.

In conclusion, pandemics characterized by high transmissibility, asymptomatic spread, or rapid surges—such as COVID-19—demand fundamentally different surveillance strategies than those used for low-incidence diseases. When policies carry legal consequences, such as regional lockdown, decisions must be grounded in high-quality, statistically sound data [24]. Probability sampling becomes crucial for tracking epidemic dynamics. Evidence-based policies, resource allocation, and public trust hinge on reliable information. Robust statistical data form a non-negotiable foundation. Without statistical rigor, volume and speed are irrelevant.

Disease-specific features directly shape sampling strategy. Logistical and ethical challenges—privacy, test evolution, nonresponse—further complicate real-time implementation. Sustained political support, inter-agency coordination, and stable funding are necessary. Yet the cost of continued reliance on biased surveillance far outweighs these obstacles and the resulting information gain justifies the additional effort. Although resource-intensive, probability-based surveillance eliminates selection bias by design and substantially reduces decision-relevant uncertainty, particularly for guiding timely interventions during pandemics. Statistical uncertainty is inherent and must be rigorously quantified, transparently communicated, and systematically incorporated into decision-making. Surveillance systems should therefore be adaptive, activating probability sampling once predefined epidemic thresholds are exceeded. Hence, for future pandemics, integrating probability sampling into preparedness plans is not optional—it is indispensable. Effective public health response begins with data quality.

## Ethical statement

This work is based on simulated data only. Therefore, no ethical approval is required.

## Author contributions

All authors contributed equally.

## Funding

This work was supported by the Studienstiftung des deutschen Volkes (scholarship to IS), by Trier University (institutional support), and by the MikroSim research unit FOR 2559, funded by the German Research Foundation (DFG; http://mikrosim.uni-trier.de) for data access.

## Declaration of competing interest

The authors declare that they have no known competing financial interests or personal relationships that could have appeared to influence the work reported in this paper.
