# Supplementary material for: From case counts to probability sampling: Simulation insights into pandemic surveillance
Source: Public Health Pract (Oxf). 2026 Mar 11;11:100766. doi: 10.1016/j.puhip.2026.100766 (PMC13054679; doi:10.1016/j.puhip.2026.100766)
Supplement: Multimedia component 1 [file mmc1.docx]

# Supplementary Material

## Simulation Framework

The simulation is performed on a subset of the MicroSim^1^ core data, a statistical digital twin of the German population based on the MicroSim model as a regionalized dynamic microsimulation. Owing to computational constraints, the analysis is limited to the synthetic population of private households in the districts of Trier and Trier-Saarburg, Germany. The population is treated as fixed and finite over a 52-week period, with demographic processes such as births, deaths, and migration explicitly excluded. Institutional populations are not included. This framework allows systematic comparison of alternative scenarios and supports counterfactual “what-if” analyses to derive implications for real-world population surveillance.

## Augmented Variables

We extended the simulation dataset with variables relevant for epidemic modeling: socioeconomic status (proxied by the German Index of Socioeconomic Deprivation, GISD^2^, downloaded from the Robert Koch Institute GitHub repository^3^), spatial neighborhood categories (generated via the Lavallée–Hidiroglou stratification method^4^ using Kozak's algorithm^5^), individual infection status, symptom presence, and test outcomes per week.

## Epidemic Process

Infection dynamics were simulated over 52 weeks using a municipality-specific modified susceptible-infected-removed (SIR) model, extended to allow re-infections, with the aim of generating plausible regional and overall epidemic curves rather than reproducing exact real-world trajectories. Transitions are stochastic: susceptible to infected is driven by spatial autoregressive individual risk scores, logit-transformed and logit-scaled to match the SIR flow, while infected to removed and removed to susceptible occurs with a certain probability. Time-varying coefficients enforce phase-specific shifts in associations with age group and socioeconomic status across three pandemic periods.

## Disease Realization

Figure S1 displays the simulated prevalence trajectory. Panel (a) shows overall infection dynamics, while panel (b) presents municipality-specific curves, which follow the general trend but show marked local variability. The overall prevalence begins at 0.00005245%, peaks at 5.22%, and has a median of 1.3%. Municipality-specific maxima range from 2.16% to 12.9%. Panels (c)–(f) display age-GISD-specific prevalence trajectories. Sharp declines in some subgroups reflect change points in the underlying parameters, mimicking intervention effects such as lockdowns.

## Symptoms and Test Outcomes

Symptoms were assigned stochastically via Bernoulli draws, assuming 3% symptomaticity among SARS-CoV-2 negative individuals based on GrippeWeb^6^ estimates and 50% among infected individuals. This yields an overall symptom rate near 3% that rises modestly (up to about 5.4%) as prevalence increases. Observed individual RT-PCR test outcomes were then generated using sensitivity of 98.1% and specificity of 98.7%.

Fig. S1. Simulated weekly prevalence rates across municipalities and population subgroups.


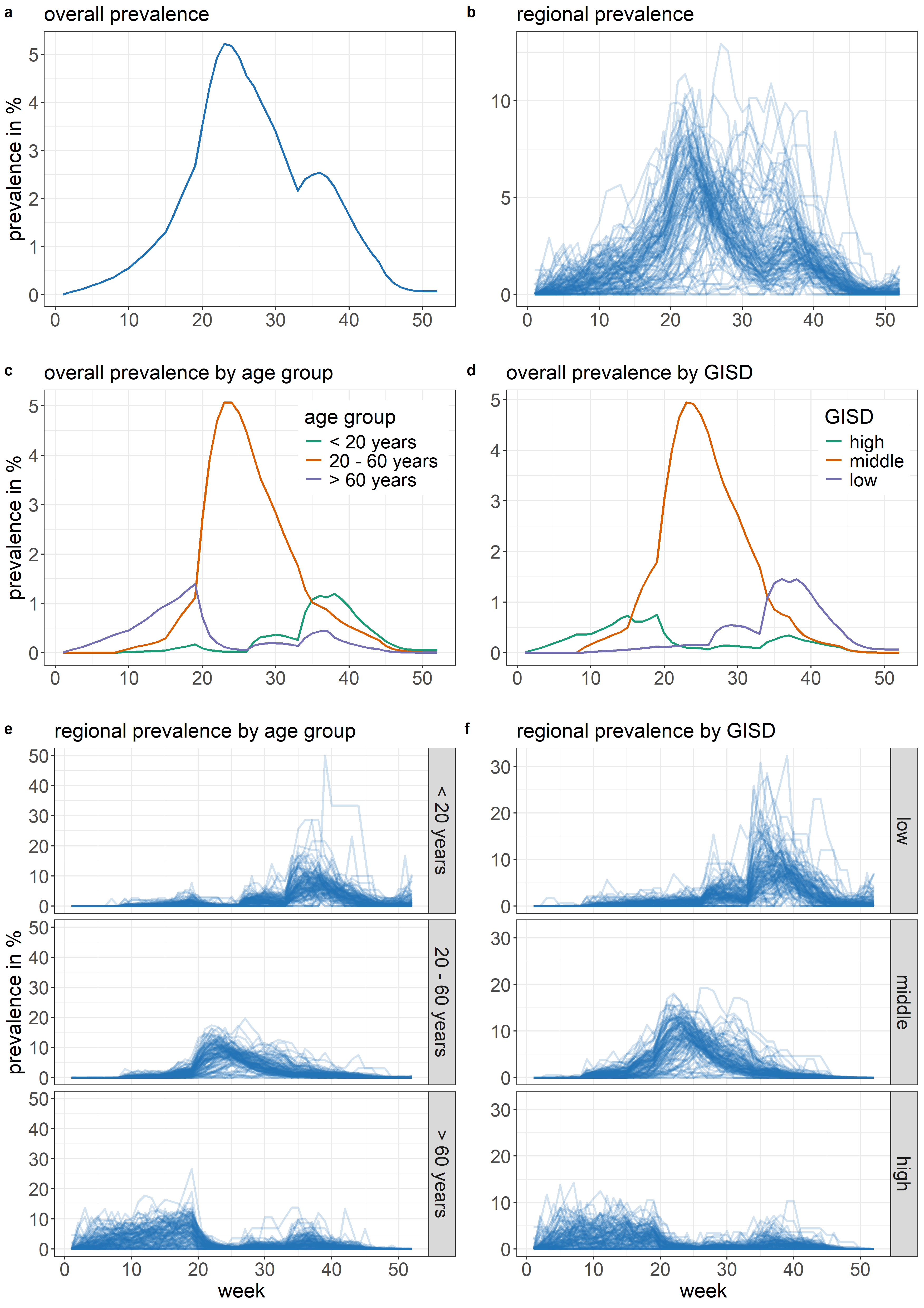


# References

1. Münnich, R, Schnell, R, Brenzel, H, et al. (2021). A Population Based Regional Dynamic Microsimulation of Germany: The MikroSim Model. methods, data, analyses. 2021;15:241-264. https://doi.org/10.12758/mda.2021.03.

2. Kroll LE, Schumann M, Hoebel J, Lampert T. Regional Health Differences – Developing a Socioeconomic Deprivation Index for Germany. J Health Monit. 2017; 2:98–114. https://doi.org/10.17886/RKI-GBE-2017-048.2.

3. Robert Koch Institut. German Index of Socioeconomic Deprivation, https://github.com/GISD-FG28/GISD/blob/master/Revisions/2022/Bund/Kreis/Kreis.csv; 2022 [accessed 06.05.2022]

4. Lavallée P, Hidiroglou M. On the stratification of skewed populations. Surv Methodol. 1988; 14:33–43.

5. Kozak M. Optimal stratification using random search method in agricultural surveys. Stat Transit. 2004; 6:797–806.

6. Robert Koch Institut. GrippeWeb-Wochenbericht, https://grippeweb.rki.de/WeeklyReports.aspx; 2022 [accessed 06.05.2022]
